# From polarity to pathology: Decoding the role of cell orientation in osteoarthritis

**DOI:** 10.1016/j.jot.2024.09.004

**Published:** 2024-10-04

**Authors:** Xiwei Fan, Louis Jun Ye Ong, Antonia RuJia Sun, Indira Prasadam

**Affiliations:** a Department of Orthopaedic Surgery, The Second Xiangya Hospital of Central South University, Changsha, China; b School of Mechanical, Medical & Process Engineering, Queensland University of Technology, Brisbane, Australia; c Centre for Biomedical Technologies, Queensland University of Technology, Brisbane, Australia; d Max Planck Queensland Centre (MPQC) for the Materials Science of Extracellular Matrices, Queensland University of Technology, Brisbane, Australia

**Keywords:** Cell polarity, Chondrocytes, Motile, Orientation, Osteoarthritis

## Abstract

Cell polarity refers to the orientation of tissue and organelles within a cell and the direction of its function. It is one of the most critical characteristics of metazoans. The development, growth, and functional tissue distribution are closely related to holistic tissue or organ homeostasis. However, the connection between cell polarity and osteoarthritis (OA) is less well-known. In OA, multiple chondrocyte clusters and tissue disorganisation can be observed in the degraded cartilage tissue. The excessive upregulation of the planar cell polarity (PCP) signalling pathway leads to the loss of cell polarity and organisation in OA progression and aetiology. Recent research has become increasingly aware of the importance of cell polarity and its correlation with OA. Several cell polarity-related treatments have shed light on OA. A thorough understanding of cell polarity and OA would provide more insights for future investigations to treat this worldwide disease.

**The translational potential of this article:**

Understanding cell polarity, associated signalling pathways, organelle changes, and cell movement in the development of OA could lead to advances in precision medicine and enhanced treatment strategies for OA patients.

## Graphical abstract

**Figure:**
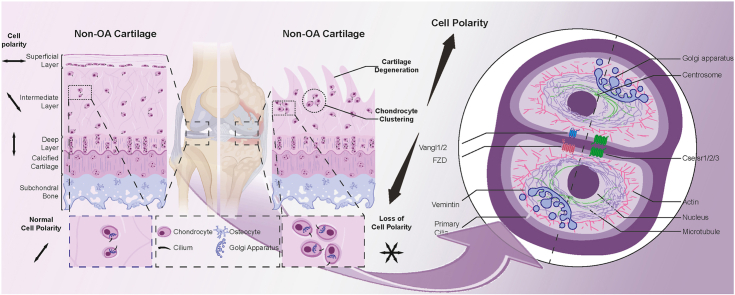

## 1 Introduction

Osteoarthritis (OA) is a chronic whole-joint disease [1] affecting all joint tissues, including degeneration of the meniscus [2], inflammation of the infrapatellar fat pad, synovial membrane, and subchondral bone remodelling [3]. Central to OA pathology is the degradation of the cartilage matrix and the loss of chondrocyte function [4,5], which are critical drivers of the disease. These pathological changes lead to thinning cartilage, loss of elasticity, roughening of the cartilage surface, and decreased joint space. As OA progresses, there is a reduction in water content within the cartilage, chondrocyte clustering, and histological disorganisation. Although OA poses a heavy health burden to society [[6], [7], [8], [9]], there is currently no disease-modifying treatment, mainly due to the limited knowledge of the disease's aetiology [9,10]. Therefore, understanding the disease's underlying mechanisms and developing potential therapies have become increasingly crucial for innovative therapeutic strategies, which are essential for mitigating the individual and societal burdens posed by OA.

In healthy articular cartilage, chondrocyte distribution varies across three distinct layers: superficial, middle, and deep. This organisation is regulated by various factors, featured by cell polarity-related genes [11]. Cell polarity is essential for maintaining tissue architecture and function, particularly in multicellular organisms. Many developing tissues can exhibit multiple forms of morphological polarisation simultaneously or in succession, though how they switch and rely on each other is less well studied [12]. Planar cell polarity (PCP) and apical-basal polarity (ABP) are two distinct aspects of cellular polarity that play vital roles in multicellular organisms [13,14]. ABP refers to the organisation and orientation of cells, mainly in epithelial tissues [15], where cells have distinct "apical" and "basal" surfaces that are asymmetric in organelle distribution, movement and function. This polarity is crucial for adequately functioning, which lines surfaces and cavities throughout the body, including the skin, lungs, and digestive tract. However, no research has been reported on ABP and cartilage in this context. In contrast, the PCP signalling pathway involved in PCP regulation is reported to be critical in chondrocyte alignment and arrangement, impacting cartilage's structural integrity and biomechanical properties [[16], [17], [18], [19], [20]]. During cartilage development, the Wnt/PCP pathway plays crucial roles in cytoskeletal reorganisation, chondrocyte stacking, and different phenotypic responses [21]. Although extensive research has been conducted on PCP dysregulation across various diseases [11], including neural tube defects [22], kidney disease [[23], [24], [25]], cancers [[26], [27], [28]], and other conditions, the role of PCP in cartilage health and pathology is still notably underexplored. Understanding the relationship between PCP and OA can aid the development of disease-modifying drugs for OA. The promising results would shed light on potential therapeutic strategies targeting cartilage-related disorders by examining the relationship between cell polarity and cartilage integrity.

## 2 Cartilage cell polarity is essential for specialised cell functions

Cell polarity is crucial for numerous cellular processes, reflecting the intrinsic differences in cell shape, structure, and organisation of cellular components [11]. These asymmetries enable cells to undertake specialised roles and maintain their unique characteristics. ABP and PCP are the main types of cell polarity essential for various cellular functions and tissue organisation. ABP refers to the asymmetrical organisation of cellular components along the apical-basal axis in cells, particularly in epithelial cells. Epithelial cells line the surfaces of organs and body cavities and serve as barriers against pathogens. ABP enables epithelial cells to have distinct apical (upper) and basal (lower) surfaces with different protein compositions and functions [15]. For example, the apical surface of epithelial cells may have microvilli to increase surface area for absorption, while the basal surface connects the cells to the underlying basement membrane [29]. PCP refers to the coordinated polarisation of cells within the plane of a tissue. This type of polarity is essential for various developmental processes and tissue organisation. PCP signalling involves the asymmetric localisation of "core" PCP protein complexes at the cell membrane, which influences the orientation of cells concerning their neighbours [25]. For instance, PCP is crucial for adequately developing hair cells in the mammalian inner ear. These cells detect sound vibrations and transmit them to the brain as electrical signals. PCP signalling helps align the hair cells in a specific direction to optimise their ability to sense sound waves [30]. Several below-mentioned factors highlight the importance of cellular polarity in facilitating seven cellular processes:

**Barrier function**: In epithelial cells, ABP is vital for providing a barrier function against pathogens [15]. This polarity ensures that the cells are appropriately oriented and adhere to each other, forming tight junctions that prevent the entry of harmful substances and microorganisms. **Cell migration**: Cell migration requires PCP, which allows cells to adhere to and detach from the extracellular matrix (ECM) [31]. This is essential for various biological processes, including wound healing, immune response, and tissue development. **Establishment of signalling domains**: Cell polarity is defined by creating segregated signalling domains in the plasma membrane and cytoplasm, which are crucial for a range of cell functions such as motility, barrier formation, and fate determination [32]. **Cytoskeleton orientation**: The structural orientation of the cytoskeleton, specifically actin filaments and microtubules, can also define cell polarity [33]. This is essential for cell migration and motility, where front-rear polarity determines the direction of migration. **Impact on cancer**: Cell polarity significantly influences cancer initiation because of the increased incidence of asymmetric division. Moreover, cells become increasingly migratory, which contributes to disease progression and facilitates metastasis [34]. **Fate determination**: Cell polarity is essential for cellular functions and developmental processes [35]. For example, fate determination may depend on preexisting cell polarity inherited from the mother cell (intrinsic) or be established after division based on exposure to a distinct signalling regime (extrinsic).

**Fig. 1:**
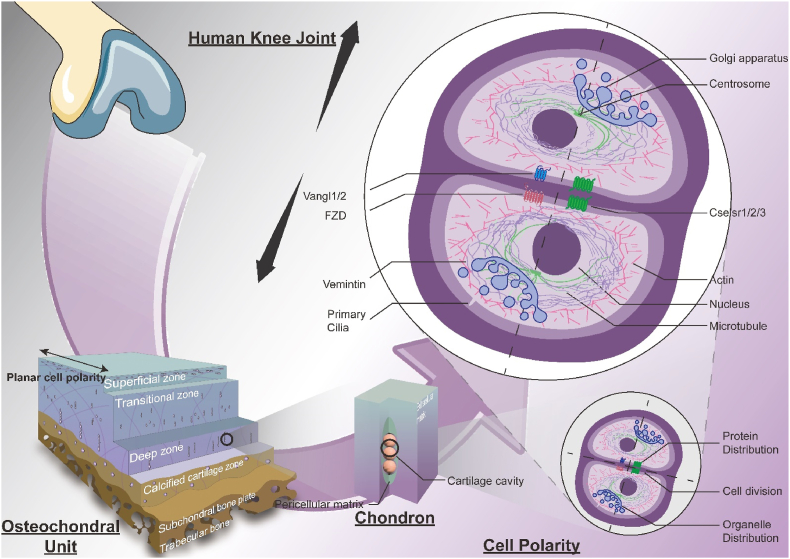
**Illustration of planar cell polarity within a typical osteochondral unit.** Cell polarity within the osteochondral units and chondrocyte arrangement of the human knee joint is crucial for joint health. Vital components of the PCP signalling pathway, such as Vangl1/2, FZD and Cselsr, are critical for maintaining cell orientation and the directional interactions amongst cells. The integrity of cytoskeletal elements like microtubules and actin filaments is essential for the proper division of cells and organisation of the matrix, with their dysfunction linked to the progression of osteoarthritis. The positioning of the Golgi apparatus and centrosome dictates the direction of cell polarity, with alterations in their orientation and location closely associated with the advancement of osteoarthritis. In summary, there's a complex interplay between cell polarity and osteoarthritis, with a deeper understanding of the molecular and cellular dysfunctions providing a theoretical foundation for the research and development of Disease-Modifying Osteoarthritis Drugs (DMOADs). The figure was created with Biorender.com.

Various papers reported that PCP is vital in maintaining chondrocyte cell direction and functional asymmetry, which are crucial for cartilage homeostasis [36,37]. Different markers have been identified as essential components for this context, as summarised in Fig. 1, which are addressed in the following sections.

## 3 The role of PCP signalling pathway and core proteins in chondrocyte function and OA progression

OA is a multifaceted and dynamic condition that arises from an imbalance between the preservation and degradation of joint tissue integrity. Among the molecular pathways involved, the Wnt signalling pathway has gained attention due to its significant role in OA pathogenesis [[38], [39], [40], [41]]. The Wnt signalling pathway is divided into three main branches: the Canonical Wnt pathway (Wnt/β-catenin pathway), the PCP pathway, and the Wnt/Ca^2+^ pathway [21,[42], [43], [44]].

The PCP pathway, a non-canonical branch of Wnt signalling, is initiated by binding Wnt proteins to Frizzled receptors. Unlike the canonical pathway, the PCP pathway does not involve β-catenin-mediated gene transcription. Instead, it regulates the cytoskeleton and controls the polarity of cells within the plane of a tissue, playing a crucial role in tissue morphogenesis and the coordinated orientation of cells and their structures [11,[45], [46], [47], [48], [49]]. The pathway's core proteins—Frizzled (Fzd), Dishevelled (DVL), Van Gogh (Vangl), Flamingo (CELSR), Prickle, and Diego—are essential for establishing and maintaining PCP [50], as shown in Table 1. These proteins interact with each other and with other cellular components to regulate the cytoskeleton and control cell polarity, with disruptions potentially leading to defects in PCP and associated developmental abnormalities [50].

**Table 1:** PCP proteins in Drosophila and Human.

Function	Mammalian name (human/mouse symbol)	Description	Biological Process	Cellular Location
Core PCP proteins	VANGL PCP Protein 1 (VANGL1/Vangl1)	Four-pass transmembrane protein	Cell polarity regulation	Plasma membrane
	VANGL PCP Protein 2 (VANGL2/Vangl2)			
	Prickle PCP Protein 1 (PRICKLE1/Prickle1)	Membrane-associated protein; Van Gogh binding partner	Cell polarity regulation	Cytoplasmic side of plasma membrane
	Prickle PCP Protein 2 (PRICKLE2/Prickle2)			
	Prickle PCP Protein 3 (PRICKLE3/Prickle3)			
	Prickle PCP Protein 4 (PRICKLE4/Prickle4)			
	Frizzled Class Receptor 3 (FZD3/Fzd3)	Four-pass transmembrane protein; Wnt family receptor	Wnt signalling pathway	Plasma membrane
	Frizzled Class Receptor 6 (FZD6/Fzd6)			
	Dishevelled Segment Polarity Protein 1 (DVL1/Dvl1)	Membrane-associated protein; Frizzled binding partner	Wnt signalling pathway	Cytoplasm
	Dishevelled Segment Polarity Protein 2 (DVL2/Dvl2)			
	Dishevelled Segment Polarity Protein 3 (DVL3/Dvl3)			
	Ankyrin Repeat Domain 6 (ANKRD6/Ankrd6)	Membrane-associated protein; Frizzled binding partner	Wnt signalling pathway	Cytoplasm
	Inversin (INVS/Invs)			
	Cadherin EGF LAG Seven-Pass G-Type Receptor 1 (CELSR1/Celsr1)	Nonclassical cadherin; Frizzled and Van Gogh binding partner	Cell adhesion	Plasma membrane
	Cadherin EGF LAG Seven-Pass G-Type Receptor 2 (CELSR2/Celsr2)			
	Cadherin EGF LAG Seven-Pass G-Type Receptor 3 (CELSR3/Celsr3)			
Upstream proteins & Wnt ligands	FAT Atypical Cadherin 1 (FAT1/Fat1)	Atypical cadherin protein; Dachsous binding partner; Four-jointed substrate	Cell adhesion	Plasma membrane
	FAT Atypical Cadherin 2 (FAT2/Fat2)			
	FAT Atypical Cadherin 3 (FAT3/Fat3)			
	FAT Atypical Cadherin 4 (FAT4/Fat4)			
	Dachsous Cadherin-Related 1 (DCHS1/Dcsh1)	Cadherin-related protein	Cell adhesion	Plasma membrane
	Dachsous Cadherin-Related 2 (DCHS2/Dcsh2)			
	Four-Jointed Box Kinase 1 (FJX1/Fjx1)	Golgi resident kinase	Kinase activity	Golgi apparatus
	Wnt Family Member 1 (WNT1/Wnt1)	Wnt family secreted ligand; not known to regulate vertebrate PCP	Wnt signalling pathway	Extracellular space
	Wnt Family Member 9A/B (WNT9A/B/Wnt9a/b)	Wnt family secreted ligand; known to regulate vertebrate PCP	Wnt signalling pathway	Extracellular space
	Wnt Family Member 5A/B (WNT5A/B/Wnt5a/b)	Wnt family secreted ligand; known to regulate vertebrate PCP	Wnt signalling pathway	Extracellular space
	Wnt Family Member 4 (WNT4/Wnt4)	Wnt family secreted ligand; proposed to regulate vertebrate PCP	Wnt signalling pathway	Extracellular space
Downstream effector proteins	Inturned PCP Protein (INTU/Intu)	Guanine nucleotide exchange factor; ciliogenesis regulator	Ciliogenesis	Cytoplasm
	Fuzzy PCP Protein (FUZ/Fuz)	—	Ciliogenesis	Cytoplasm
	WD Repeat Containing PCP Effector (WDPCP/Wdpcp)	Cytoplasmic WD40 repeat protein; ciliogenesis regulator	Ciliogenesis	Cytoplasm
	Cilia And Flagella Associated Protein 126 (CFAP126/Cfap126)	The ciliogenesis regulator, also known as Flattop	Ciliogenesis	Cilia
	Scribble PCP Protein (Scrib/SCRIB)	Scaffolding protein; apical-basal polarity regulator; Vangl binding partner	Cell polarity regulation	Cytoplasm

**Fig. 2:**
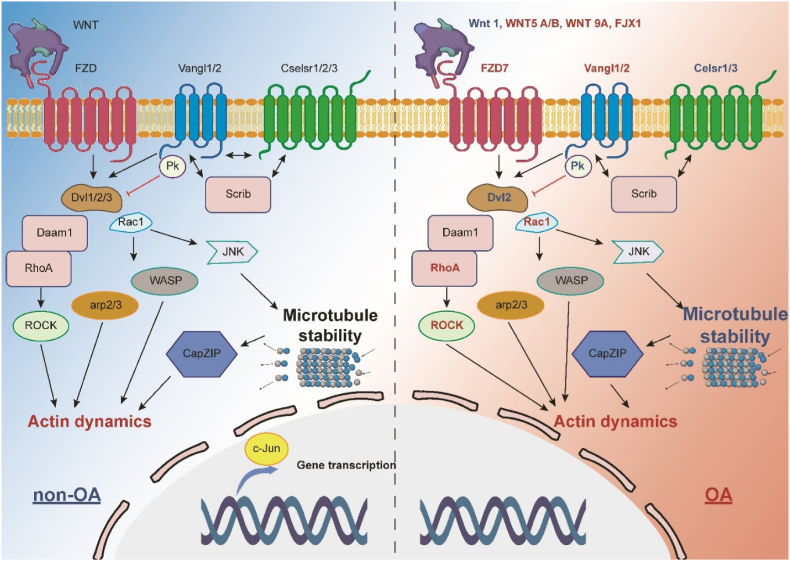
**Alterations in the WNT/PCP Signalling Pathway During the Progression of OA.** The illustration demonstrates that during the advancement of OA, there's an increase in the expression of upstream WNT5a/b and WNT9a. This activity initiates a cascade upregulation effect, causing an upregulation in the WNT/PCP signalling pathway via key PCP and downstream effector proteins. Subsequently, this leads to an escalation in chondrocyte hypertrophy and a disruption in microtubule stability, leading to polarised cell division proliferation and chondrocyte clusters. The red colour on the right figure shows upregulation in the OA condition, and the blue colour shows downregulation in the OA condition. The figure was created with Biorender.com. (For interpretation of the references to color in this figure legend, the reader is referred to the Web version of this article.)

Recent research has suggested a significant role of the Wnt/PCP pathway in OA progression [21,44,51]. While the pathway is crucial for cartilage formation and the differentiation and enlargement of cartilage cells during development, its role in mature cartilage is more complex and sometimes contradictory. Several studies have indicated that excessive activation of Wnt/PCP signalling could lead to cartilage damage and contribute to OA development, making PCP pathway inhibitors potential therapeutic targets for OA treatment. Detailed illustrations are shown in Fig. 2.

### 3.1 PCP core proteins in OA progression

During OA progression, multiple PCP core proteins show altered expression patterns. Fzd receptors, involved in the Wnt and other signalling pathways, may be implicated in OA when bound to Wnt proteins and activating the PCP pathway. For instance, FZD3 and FZD6 were found to be downregulated in bone marrow-derived mesenchymal stem cells (BMSCs), which are chondrocyte progenitor cells, from OA patients [52]. Conversely, FZD7 is upregulated in human OA cartilage [53]. Vangl proteins, including Vangl1 and Vangl2, are crucial for the asymmetric arrangement of planar polarised cells, contributing to endochondral osteogenesis and limb elongation [54,55]. In OA, Vangl2, which is asymmetrically positioned along the proximal-distal (P-D) axis in newly differentiated chondrocytes, is upregulated in an OA mimic environment induced by IL-1β [56].

Other core proteins, such as DVL, CELSR, and Prickle, also show significant changes during OA. For instance, DVL2 exhibits increased methylation levels associated with OA [57]. High methylation levels in CELSR1 and CELSR3 have been reported in human OA cartilage [58]. A decrease in PRICKLE1 has also been observed in OA [59,60].

### 3.2 Upstream and downstream proteins in PCP signaling and OA

Upstream proteins, including Wnt1, Wnt5a/b, Wnt9A, and FJX1, play significant roles in OA progression. The role of Wnt1 in OA is controversial, with reports of higher methylation during OA progression [57] and heterozygous WNT1 mutation being associated with less age-related cartilage deterioration [61]. Wnt5A, another crucial ligand, activates the PCP pathway. However, its expression varies, being downregulated in BMSCs from OA patients [52] but upregulated in chondrocytes treated with IL-1β [56], as well as upregulated in cartilage tissue in OA condition [62]. Wnt9A is another important protein, with increased expression reported in human hand OA cartilage and animal studies showing that Wnt9A knockout leads to joint degeneration [63,64]. FJX1 is also upregulated in OA cartilage compared to non-OA patients, as evidenced by whole-genome microarray analysis [62].

Downstream effectors of the PCP pathway, such as Rho-associated coiled-coil kinase (ROCK), are also implicated in OA. ROCK is upregulated in OA and correlated with chondrocyte hypertrophy [18]. Other small GTPases, including Rho, Rac, and Cdc42, initiate chondrocyte hypertrophy by interacting with Sox9 and Runx2 [65]. Additionally, c-Jun N-terminal kinases (JNKs), downstream effector Rac Family Small GTPase 1 (Rac1), play central roles in stress signalling pathways, influencing gene expression, neuronal plasticity, regeneration, cell death, and the regulation of cellular senescence in cartilage [66,67]. Specifically, Wnt16 stimulates PCP/JNK and crosstalks with the mTORC1-PTHrP pathway to inhibit chondrocyte hypertrophy [67]. In summary, the reactivation of developmental genes characterised by hypertrophic chondrocytes is associated with the loss of PCP. Detailed changes are presented in Table 2.

**Table 2:** Reported PCP signalling-related change in physiological and OA chondrocytes.

Role	PCP Protein	Role in Humans	Physiological Role in Chondrocytes	Molecular Linkage with OA
Core PCP proteins	Fzd3	Involved in Wnt/PCP signalling	Regulates chondrocyte polarity and differentiation	Downregulated in OA, impairing chondrocyte function [52]
	Fzd6	Involved in Wnt/PCP signalling	Maintains cartilage structure and chondrocyte polarity	Downregulated in OA, associated with loss of cartilage integrity [52]
	Fzd7	Participates in Wnt signalling	Supports cartilage formation and maintenance	Upregulated in OA, linked to cartilage degradation [53]
	Vangl2	Crucial in cell polarity and PCP	Asymmetrically positions in chondrocytes for proper cartilage development	Upregulated in OA, contributing to cartilage damage [56]
	Dvl2	Central to Wnt/PCP signalling	Mediates chondrocyte differentiation and cartilage development	Increased methylation in Dvl2 correlates with OA progression [57]
	Celsr1	Involved in PCP signalling	Maintains chondrocyte arrangement and cartilage integrity	High methylation levels associated with OA progression [58]
	Prickle1	Interacts with core PCP proteins to regulate polarity	Ensures proper chondrocyte polarity and cartilage formation	Decreased expression contributes to chondrocyte dysfunction in OA [59,60]
Upstream proteins & Wnt ligands	Wnt4	Activates Wnt/PCP signalling	Promotes chondrocyte differentiation and cartilage homeostasis	Conditional WNT4 Knockout in the mice mesenchymal cell leads to spontaneous OA [39]
	Wnt5A/B	Activates Wnt/PCP signalling in various tissues	Promotes chondrocyte differentiation and cartilage homeostasis	Upregulated in OA, exacerbating cartilage damage [62]
	Wnt9A	Activates Wnt/PCP signalling	Essential for joint development and cartilage maintenance	Conditional WNT9A Knockout in the mice mesenchymal cell leads to spontaneous OA [39]
	Wnt16	Activates Wnt/PCP signalling	Wnt16 activates PCP/JNK and crosstalk with the mTORC1-PTHrP pathway to inhibit chondrocyte hypertrophy	Wnt16 deficiency mice induce OA progression, and Wnt16 adenovirus intra-articular injection alleviates OA progression [67]
Downstream proteins & Wnt ligands	ROCK	The downstream effector of RhoA that phosphorylates and activates LIM kinase, which subsequently phosphorylates cofilin, inhibiting its actin-depolymerizing function	Regulates chondrocyte shape and mechanical stress response	Upregulated in OA, promoting chondrocyte hypertrophy and cartilage breakdown [65]

## 4 Multiple PCP-related organelles in close relationship with the OA progression

### 4.1 Microtubule

The cytoskeleton coordinates the internal composition of cells, including actin microfilaments, tubulin microtubules, and intermediate filaments. In addition to cell surface proteins that aid in the formation and maintenance of primary cilia in chondrocytes, the dynamics of microtubules and actin filaments have been demonstrated to play a role in the polarity and organisation of chondrocytes [68], and this leads to the rearrangement of the cytoskeleton and changed level in transcription, subsequently activating transcription-related proteins in OA.

Microtubules play a pivotal role in PCP signalling, essential for cellular alignment and cartilage matrix organisation, by regulating the orientation of cells within the plane of a tissue. Microtubule dynamics are integral in coordinating cell positioning and alignment during tissue development [69], interacting with two molecular modules in PCP signalling: the Ft/Ds/Fj system and a core complex including Fzd and Dsh. By organising the microtubules, the Ft/Ds/Fj module provides directional cues to the core complex, aiding cell polarisation within epithelia. In OA, PCP signalling disruptions lead to irregular cell alignment and cartilage matrix disorganisation. Impaired microtubule function can compromise PCP signalling, exacerbating OA pathogenesis. This relates to the Wnt/PCP pathway's role in OA, particularly involving proteins like Vangl2, which is vital for chondrocyte orientation and alignment in cartilage development. In OA, Vangl2 influences matrix metalloproteinases (MMPs) and cartilage gene expression, which is crucial for joint integrity. Vangl2's involvement in OA is underscored by its interaction with Wnt5a, part of the non-canonical Wnt pathway, which is pivotal in cartilage inflammation. Wnt5a activates catabolic signalling in chondrocytes, leading to an increase in inflammatory cytokines, chemokines, and MMPs and a decrease in key cartilage components like type II collagen and aggrecan. Thus, disruptions in microtubule function that impair PCP signalling, particularly involving key proteins like Vangl2, contribute to OA's pathogenesis. This occurs through enhancing inflammatory processes, cartilage matrix degradation, and chondrocyte function alteration [56].

Furthermore, microtubules are involved in regulating chondrocyte behaviour and cartilage homeostasis. They provide structural support to the chondrocytes and facilitate the transport of key signalling molecules and nutrients within the cells [70]. Microtubules also participate in the organisation of the cytoskeleton and influence cellular processes such as cell division, migration, and differentiation.

Microtubules are also deeply involved in OA induced by aberrant mechanotransduction and post-modifications via influencing microtubule stability. In normal chondrocytes, the matrix's rigidity affects the microtubule network's polarisation during cell migration [71]. HDAC6, a histone deacetylase, is closely related to microtubule changes in OA pathogenesis [72,73]. Deacetylase activity of HDAC6 leads to an unstable chondrocyte microenvironment, and it was recently found to increase during OA progression in the mouse model [72]. On the contrary, by stabilising the microtubule by acetylation, a recent study reported an increase in hyaline cartilage content in a rat cartilage defect model [74], which reflects the importance of microtubule change and its potential therapeutic role in OA. However, currently, there is no direct link between microtubule stability change and PCP change reported in the OA field.

Microtubules play a critical role in cell division and differentiation, and disruptions to this process can lead to abnormal cell behaviour or population imbalances in joint tissues [75]. Similarly, changes in PCP signalling could influence the differentiation of progenitor cells in the joint, possibly leading to an imbalance between cartilage-degrading and cartilage-forming cells, contributing to OA.

In summary, microtubule stability, dynamics, and organisation alterations have been observed in chondrocytes. These changes can impact cell viability, proliferation, and matrix synthesis. Additionally, abnormal microtubule organisation may disrupt mechanotransduction, which is crucial for maintaining chondrocyte phenotype and cartilage integrity.

### 4.2 Actin

Actin is another essential cytoskeleton that interacts with other organelles to maintain all eukaryotic organisms' cell shape and polarity [76]. Multiple types of actin exist, including α-actin (mainly in Muscle cells), β-actin (mainly in the cytoplasm of non-muscle cells), and γ-actin (mainly found in the cortical and lamellar regions). Actin exists either as monomers (G-actin) or in a polymerised form (F-actin), with F-actin capable of forming stress fibres (SFs). Actin is crucial in various cellular processes, including cell division, endocytosis, and migration. The polymerisation and depolymerisation of actin are regulated by several proteins, such as small GTPases like RhoA, Rac1, and Cdc42, as well as downstream effector proteins like ROCK, LIM domain kinase (LIMK), and cofilin [77]. These regulatory proteins modulate the actin polymerisation state, influencing cells' mechanical properties and motility. Meanwhile, the stress fibres' orientation determines the cell's contractile properties [78]. Several signalling pathways have been proven to regulate actin polymerisation to further influence cell polarity. For example, IL-1β increases F-actin content through RhoA, affecting cell morphology [79]. IL-6 and IL-8 have been shown to regulate actin polymerisation via the Rho-ROCK pathway [80,81].

In OA, the expression of actin-polymerizing proteins (e.g., cofilin-2 and gelsolin) increases in chondrocytes while depolymerising proteins (e.g., destrin and cofilin-1) decrease [82] This shift results in overall increased actin polymerisation, which subsequently affects cellular mechanics and phenotype [81]. A recent study has also shown that Superficial and deep zone articular chondrocytes show distinct differences in actin polymerisation status and the expression of actin-associated molecules in bovine metacarpal-phalangeal joints [83]. Adveverin is an actin-binding protein. Deleting Adseverin (Adseverin−/−) in mice impairs chondrocyte function by reducing the expression of F-actin, resulting in stiffer cartilage, reduced hyaline content, and increased calcified cartilage thickness. Consequently, these alterations lead to increased severity of osteoarthritis in Adseverin−/− mice subjected to OA surgery induction [84].

### 4.3 Primary cilia

Primary cilia are tiny hair-like structures that protrude from the surface of nearly all mammalian cells. The arrangement of primary cilia has been demonstrated to reflect cell polarity in various tissues [[85], [86], [87]]. Historically, they were considered vestigial organelles, but current scientific understanding portrays them as complex signalling centres, including Sonic Hedgehog (Shh), Wingless/Int (Wnt), and platelet-derived growth factor (PDGF) pathways [88].

The primary cilia have been shown to have a specific direction in the cartilage and are always pointing far away from the articular surface of the articular cartilage or the centre of the columnar cells in the growth plate [[89], [90], [91]]. The deep zone of articular cartilage, located close to the subchondral bone, is characterised by hypertrophic chondrocytes that contribute to extracellular matrix secretion [92]. In this zone, the main cilia point in various directions and do not follow a uniform pattern [91,93]. Primary cilia in the translational zone always have two directions, either pointing to the subchondral bone or the cartilage surface [93]. Conversely, the superficial zone is featured by the chondrocytes aligning parallel to the articular surface [94]. However, within the weight-bearing regions of the superficial cartilage, primary cilia do not align with the cell axis by constantly pointing towards the subchondral bone [93,95].

Unlike healthy cartilage, in the case of osteoarthritic cells, primary cilia reorient towards the core of anomalous chondrocyte clusters [96,97]. Although no study directly reveals the underlying link between primary cilia, PCP, and OA—evidence suggests that it plays an important role in PCP and undergoes significant changes during OA progression. Therefore, the linkage exists but is underestimated. OA is reported to be closely related to primary cilia through mechanobiological and other mechanisms [98,99]. Meanwhile, knocking out mechanosensing ion channel TRPV4 reduces age-related OA [99]. As is well known, upregulated matrix metalloproteinase 13 (MMP-13) levels function as the pivot factor by degenerating the collagen II in OA [100,101]. According to recent research, there are several ways to stimulate the expression of MMP-13 in cartilage tissue. On the one hand, IL-1 will stimulate nucleoprotein 1 (NUPR1) and cause the increased expression of MMP-13 [101]. Interestingly, Wann et al. demonstrated that protein kinase A (PKA) stimulated by IL-1beta can increase cilia length, which suggests that IL-1β induced primary cilia elongation, thus leading to inflammatory chemokine release [102].

Primary cilia can also impact the signalling pathways in OA. It was reported that primary cilia also play a role in the TGF-1/HTRA1/DDR-2 axis and the Notch signalling pathway, affecting the expression of MMP-13 [101]. Another study by knocking out mouse ciliary motor protein Kif3a showed that disturbed cilia function leads to dysregulation in MMP-13 levels [103]. However, whether there is a potential link between the expression of MMP-13 and ADAMTS-5 and the ciliary Ihh pathway is still controversial. Studies have shown that the expression of MMP-13 and ADAMTS-5 does not depend on Ihh, nor is it associated with IL-1β [104]. Another study showed that cilia also lead to increased levels of MMP-13 and MMP-1 through another signalling pathway. This study showed that the cyclical stress on human chondrocytes could upregulate the expression of Cbp/p300 interaction transactivator and ED-rich tail 2 (CITED2) and inhibit the expression of MMP-1 and -13. Downregulation of IFT88 reduces the expression of CITED2 but upregulates the expression of MMP-1 and MMP-13 [105]. In addition, studies indicated that during the progression of OA, the main cilia-related factor, TGF-3, is involved in anabolism, and the PIEZO ion channel is involved in catabolism [99,106,107]. The above studies indicated that primary cilia were indirectly related to PCP and OA progression. More research should be performed to reveal the link between primary cilia and PCP in the OA context.

### 4.4 Golgi apparatus

The Golgi apparatus is a highly polarised organelle located near the nucleus and involved in intracellular transportation. Processes proteins delivered from the endoplasmic reticulum (ER) and acts as a microtubular organising centre (MTOC) in mammalian cells. It is also a vital organelle involved in protein processing and secretion, which has been implicated in OA and its connection to PCP [108]. However, as there are limited papers focusing on Golgi apparatus change during OA progression, there is no direct evidence proving that the Golgi apparatus is involved in PCP change. However, there is some indirect connection between Golgi apparatus change and PCP change.

The Golgi apparatus is reported to undergo significant morphological changes during OA progression. Juan et al. [108] reported a modification of the Golgi apparatus in the OA rat samples. Moreover, they also observed the bubble-like structure in the Golgi with the morphologic change of the nucleus, which is believed to be the Golgi fragmentation during OA progression. It correlates with OA chondrocytes' apoptosis, as validated in human samples [109].

Concerning PCP, the Golgi apparatus participates in the trafficking and localising of PCP signalling molecules [110]. PCP signalling regulates the orientation and alignment of cells within a plane, including chondrocytes in cartilage. The Golgi apparatus ensures the proper delivery of PCP signalling components to the plasma membrane, where they initiate cellular responses involved in tissue development and maintenance [111]. As stated previously, multiple PCP-related proteins were found to change during OA progression. Disruptions in Golgi-dependent trafficking mechanisms can impair PCP signalling, contributing to OA's aberrant cell alignment and cartilage organisation.

The Golgi apparatus is also involved in the post-translational modification of proteins. Glycosylation, a process occurring within the Golgi, can impact the activity and stability of PCP molecules. Alterations in Golgi-mediated glycosylation pathways may influence the function of proteins, ultimately affecting cell polarity in OA. Several studies proved that glycosylation changes OA progression in human samples [[112], [113], [114]]. However, whether the Golgi apparatus is involved in the post-modification of core PCP proteins remains to be further studied. In summary, whether the Golgi morphological and functional changes are involved in the chondrocyte and PCP changes remains to be investigated in future studies.

### 4.5 Centrosome

**Fig. 3:**
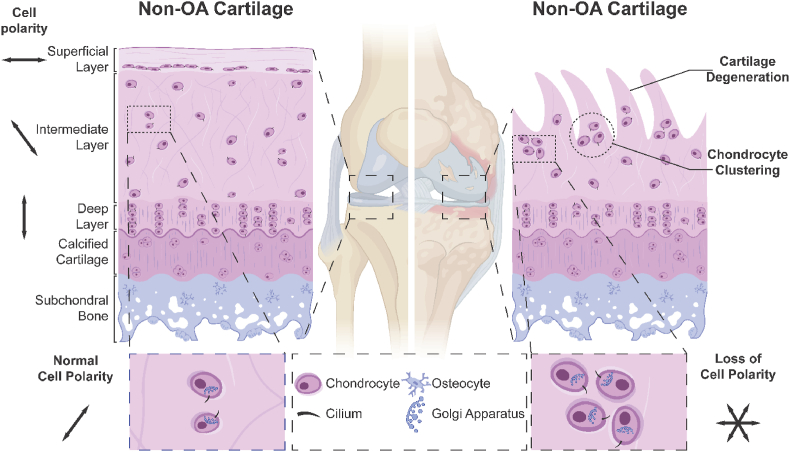
**Alteration of Cell Polarity in OA Progression.** The figure illustrates the comparison between healthy and OA cartilage, highlighting the changes in cell polarity during OA progression. The non-OA cartilage is clearly defined as superficial, intermediate, and deep layers, where chondrocytes are well-organized and maintain their polarity, as shown by the orientation of their cilia and Golgi apparatus. The subchondral bone and calcified cartilage layers exhibit a polarised structure, providing stability and support to the cartilage. In contrast, in OA cartilage, cartilage degeneration leads to cartilage delamination, and chondrocytes lose their organised structure, forming abnormal clusters. The cell polarity in cartilage is also arranged properly in normal cartilage, parallel to the joint surface at the superficial layer, leaning at the intermediate layer and longitudinal to the joint surface at the deep layer, but in OA chondrocytes, the cell polarity is misaligned. The loss of cell polarity disrupts the chondrocyte's normal function, contributing to the progression of OA. The figure was created with Biorender.com.

The centrosome comprises a centriolar core surrounded by a pericentriolar material essential for microtubule formation. The orientation of cell division, cell migration, and the polarised immunological response of lymphocytes are all determined by centrosome placement. The nucleus and the centrosome both strive for a central position in the cell, which means that the nucleus also competes with the centrosome to some extent [115]. The centrosome is connected to the nucleus and is located around 0.2 μm distant from the nuclear envelope [116]. When the nucleus has a multilobulated structure, such as in neutrophils, the centrosome is inside the nucleus courtyard [117,118]. The mutual arrangement of the nucleus and centrosome was investigated using a previously developed model of round and triangular-shaped single cells cultured on a micropatterned substrate [119]. The link between the nucleus and the centrosome weakens as nocodazole disrupts microtubules, thus increasing the distance between the two organelles. The nucleus was shifted away from the central position, and the nucleus-centrosome distance increased when latrunculin B disrupted actin filaments. The nucleus-centrosome distance rose considerably in round-shaped cells with reduced nuclear membrane lamin A, most likely due to the cytoskeletal components’ inability to connect to the nucleus. However, until now, no relevant studies have directly reported the increase of centrosome change during OA progression. Detailed cell polarity changes of OA chondrocytes are illustrated in Fig. 3.

## 5 Chondrocytes division, mobility, and PCP change

### 5.1 Polarised cell division of the growth plate is crucial for the development and growth

Polarised cell division refers to the asymmetric segregation of cellular components during cell division, which results in two daughter cells with distinct fates. This process is essential for maintaining tissue homeostasis and organisation. Abnormalities in polarised cell division can disrupt tissue architecture and contribute to OA development [48].

Two basic cell characteristics, including oriented cell division and cell rearrangement, play an important role in tissue growth [120,121]. By orienting the division axis in a specific direction, orienting cell division has two primary purposes: First, they can drive the body axis to elongate, such as endochondral ossification that leads to limb lengthening [122]. Secondly, they are very important for cell diversity. For example, the asymmetric division determines whether the cells become stem cells or differentiated cells, just like the phenomenon observed in the spermatogenesis of Drosophila [123]. Cell rearrangement involves the exchange of substances between cells, which can occur through different mechanisms, including connection remodelling. The adhesion connection between cells shrinks in one direction and extends in an orthogonal direction, as seen in the epithelial tissue [124]. The second example is the embedment of mid-lateral cells during embryonic development. A typical case is the chordal cells during the formation of Xenopus gastrula [125]. All the above-mentioned cellular properties can establish and maintain polarised cellular tissues, leading to body elongation [126,127]. Another research employing clonal analysis indicated that an individual precursor cell of cartilage can generate single and multiple-column clones, facilitated by mechanisms such as directed cell division and rearrangement. The typical tissue structure is created when a single cell undergoes a process resembling a pivot interaction between sister cells. In the case of multiple-column clones, N-cadherin, a protein, accumulates in the division groove following the split of the middle and outer compartments. Subsequently, one sibling cell pivots around the other's axis, forming multiple column clones. Further analysis indicated that Planar Cell Polarity (PCP) signalling allows these cells to rotate along the axis of limb growth, facilitated by this N-cadherin-mediated connection [36].

### 5.2 Loss of oriented cell division and OA progression

Chondrocytes, the cells found in cartilage, respond to mechano-compression as a part of their normal physiological function, allowing cartilage to absorb mechanical forces and maintain its structure. In the deep layer of cartilage, chondrocytes are aligned to resist compressive forces efficiently. At the same time, in the superficial zone, they remain parallel to the surface to accommodate shear forces and facilitate smooth joint movement. This orientation and response to mechanical stress are crucial for joints' overall health and functionality, and scientists attribute this to the interphase cell's long-axis sensing [128]. However, using clonal analysis, Li et al. [36] found that PCP signals enable cells to rotate in the direction of limb elongation through this N-cadherin-mediated coupling. Inhibition of FZD7 signalling disrupts directed cell division but does not disrupt cell pivotal behaviour. In contrast, activation of FZD7 disrupts directed cell division and cell pivot behaviour. Further analysis showed that too much membrane-bound PCP component could block the pivotal behaviour. The downregulation of cadherin inhibits both sister cell binding and its pivotal behaviour.

The chondrocyte cluster is one of the most significant features observed in OA [129]. The loss of spatial organisation and formation of clusters indicated the reactivation of proliferation capacity and loss of PCP [130]. Previous studies have found multiple markers expressed in chondrocyte clusters, including Stem cell markers [131], cytokines and growth factors, ECM and ECM degrading enzymes, cell death, and progenitor cell markers. However, the direct interconnection between PCP and polarised cell division in OA remains to be further researched.

### 5.3 Loss of polarised cell migration and OA progression

Polarised cell migration is the coordinated and directional migration of cells within a tissue, essential for maintaining normal joint function [132]. In a healthy joint, polarised cell migration contributes to properly organically maintaining cartilage, enabling smooth articulation and load distribution. For a long time, chondrocytes were considered stationary cells, resting in the cartilage cavity, keeping a spherical shape throughout the depth of the tissue, and maintaining a specific direction within a particular cartilage area. That is mainly due to the water-swelling properties of the tissue and the strengthening of its high-tension collagen network [133]. The cartilage ECM is constantly under pressure. The mechanisms above make it more difficult for chondrocytes to achieve cell migration under physiological conditions [[133], [134], [135]]. However, recently, various labs have observed the movement of cartilage cells, or chondrocytes, in response to multiple stimuli in both 2D and 3D settings. This range of incentives, which can prompt chondrocytes to move, includes bone morphogenetic factors [136], hepatocyte scatter factor [137], urokinase plasminogen activator [138], insulin-like growth factor-I (IGF-I) [139], transforming growth factor-β [139], platelet-derived growth factor [140], and fibroblast growth factor [141,142]. Moreover, chondrocytes can relocate towards several components of the cartilage matrix, such as hyaluronic acid [142,143] or its sulphated form, fibronectin [138,139], fibrin [144], collagen I [138], and even towards cathode at the electric field [145]. Studies using time-lapse video microscopy have demonstrated that chondrocytes possess adequate mobility, though their speed and directionality are somewhat lower than other cell types [133].

No direct connection exists between the polarised cell migration change and PCP change in the OA context at the current stage. However, in recent years, growing evidence supports the reactivation of chondrocyte motility during the progression of OA. In a study by Kouri et al. OA cartilage tissue showed cell aggregation and the formation of cell clumps on the superficial layer and surface of damaged cartilage [146]. The above studies also proved the changes in the cytoskeleton arrangement in the progression of OA through the presence of many filopodia and primary cilia. The investigation believes that these data indicate that cartilage cells may actively move to the injured area. In addition, another study showed that when suffering an injury, chondrocytes or cartilage progenitor cells migrate to the injury site and repair the injury by compensatory secretion of ECM [133]. This article speculates that chondrocytes can create channels for their migration by expressing proteolytic enzymes and using amoeba migration [133]. Different research delineated how cartilage precursor cells differentiate and are recruited under the guidance of synovial mesenchymal stem cells to aid in cartilage repair. By comparing the chondrocyte morphology of in normal and OA tissue and grading them by severity, the increased observation of cilia, centrioles, and filopodia in and near the clusters below and near the OA cartilage fibrillation area indicated that they might restore motor function in the diseased condition [146,147]. Studies have shown that small cytoplasmic protrusions are detected between some pairs of chondrocytes in the surface area of mature rabbit articular cartilage. This suggests that the recovery of the motor function restoration may also be related to the chondrocyte clusters, but further studies need to be done to confirm this phenomenon [129].

## 6 Therapeutic implications

### 6.1 PCP signalling pathway inhibition in OA

Although multiple pieces of evidence provide that PCP changes during OA progression [67,148], barely any paper directly reports that chondrocyte polarity maintenance is conducive to preventing or alleviating OA. However, research has demonstrated that OA can be attenuated through the non-canonical PCP signalling pathway. Recently, the WNT signalling pathway has raised much concern for its potential in treating OA [148]. Tong et al. [67] reported that Wnt16-mediated PCP/JNK and mTORC1-PTHrP pathways are activated to inhibit cartilage hypertrophy effectively. Another new study shows that Vangl, the core element of PCP, plays a pivotal role in the progression of OA. Knocking down Vangl2 can significantly mitigate in vitro OA progression by upregulating MMPs and upregulating the secretion of the ECM. It can also downregulate the proinflammatory function of Wnt5a through MAPK and NF-kB pathways.

### 6.2 PCP-related organelle conservation for OA targeted treatment

PCP-related organelles, including microtubules, primary cilia, and Golgi apparatus, experienced significant changes during OA progression; protecting chondrocyte phenotyping may be a new strategy for alleviating or reversing the disease. Multiple studies have focused on organelle phenotype maintenance in OA conditions marked by microtubule-related markers. Previous research in several types of cells, including fibroblasts [149,150], synoviocytes [151], and chondrocyte progenitors [152,153], found that the drug-induced microtube system interruption reduces collagen and proteoglycan synthesis and secretion in vitro, suggesting that microtubule regulation could be a potential strategy for treating OA. Microtube stabilisation has been found to play a major role in two pathways. The first pathway is to upregulate the TGF-β/SMAD signalling pathway to maintain cartilage cell function and to promote mesenchymal stem cells to cartilage cell differentiation. Stable microtubes also provide a stable tunnel for cells to transport and secrete extracellular matrix. Compared to the control group, the mouse model with microtube stabilisation using docetaxel showed more normal hyaluronic cartilage than the control group [154]. Lee et al. reported that the repaired tissue in the rat model of cartilage damage treated with docetaxel showed more normal hyaluronic cartilage than the control group [154]. Post-translational modification of microtubes is another potential target. The sections above mentioned that HDAC6 is closely related to microtubule acetylation, which has been shown to stabilise microtube morphology. Zheng et al. [155] reported that the overexpression of HDAC6 also leads to mitochondrial dysfunction and stimulates the generation of reactive oxygen species, thus resulting in ECM damage and degeneration. In this context, tubastatin can function as an inhibitor of HDAC6 that alleviates cartilage degradation, thus providing a promising potential treatment for OA. Recently, a newly developed thermosensitive hydrogel, explicitly designed to target fibrocartilage and carry a negative charge, has shown promise for sustained delivery of docetaxel. This advanced method has been observed to enhance the regeneration of hyaline cartilage instead of fibrous cartilage, which makes it a potential new treatment option for OA [74].

## 7 Future perspectives

The complexity of cellular behaviours driving skeletal elongation in limbs provides critical insights into chondrocyte dynamics. Previous studies have shown that clonally related chondrocytes can arrange into single or multi-columnar structures along the axis of tissue elongation, influenced by cell behaviours such as pivoting and intercalation. This arrangement is similar to cell rearrangements observed in mouse presphenoidal synchondrosis, implicating the PCP pathway in these processes [36]. The role of the PCP pathway in coordinating cell pivoting post-mediolateral division underlines its importance in cellular proliferation, and stereotypical arrangement leads to tissue elongation, particularly under OA conditions.

Recent findings from large animal models and human clinical studies have begun to bridge the gap between basic research on cell orientation and potential therapeutic strategies for OA. For example, Wnt16 has been shown in the mice preclinical study to activate the PCP/JNK pathway and engage with the mTORC1-PTHrP signalling pathway to suppress chondrocyte hypertrophy, suggesting that Wnt16 could be a promising therapeutic target for treating OA [67]. Moreover, WNT4 and WNT9A conditional knockout in mice's mesenchymal stem cells suggests that they induce spontaneous OA [39]. Prickle1 downregulation also induces OA phenotype in chondrocytes [59,60]. The above studies suggest PCP can be used as the potential treatment target. More large animal models and human clinical trials are needed to translate the insight of OA into DMOADS development.

Exploring the mechanistic links between chondrocyte behaviour in OA and the cellular dynamics observed inappropriate tissue architecture. Techniques such as single-cell RNA sequencing (scRNA-seq) and lineage tracing could be employed to study chondrocyte polarity's evolution and real-time arrangement during disease progression [156]. Additionally, innovative imaging technologies like multiphoton microscopy could provide in vivo visualisation of these cellular arrangements and interactions [157]. Further, integrating CRISPR-Cas9-mediated clonal analysis could elucidate chondrocytes' proliferative history and lineage decisions, particularly in response to PCP signalling disruptions commonly seen in OA [158]. Combining advanced genetic, molecular, and imaging techniques, this integrated approach will provide a detailed understanding of how chondrocyte polarity and arrangement contribute to OA progression. Such insights could lead to identifying novel therapeutic targets within the PCP signalling pathway, offering potential strategies to modulate these cellular behaviours to mitigate the effects of OA. These endeavours will significantly enhance our comprehension of OA's molecular and cellular mechanisms and may pave the way for innovative treatments.

## 8 Conclusion

To summarise, a complex interplay exists between PCP and the pathogenesis of OA. The critical role of cartilage cell polarity in maintaining joint health, the intricate details of the PCP signalling pathway, and the consequential changes in cartilage structure during OA progression have been highlighted. This review underscores the significance of PCP in the context of OA, revealing how disruptions in cell polarity and signalling pathways contribute to the disease's development. Furthermore, it suggests that a deeper understanding of these mechanisms could open new avenues for therapeutic interventions, potentially leading to innovative strategies to mitigate the debilitating effects of OA. Future research leveraging advanced biomedical technologies is essential further to elucidate the relationship between chondrocyte polarity and OA, providing insights that could revolutionise the treatment of this debilitating condition.

## Author contributions

Conception and design: XF, IP; Drafting of the article: XF, IP; Critical revision of the article for important intellectual content: All authors; Funding obtaining: IP; All authors take responsibility for the paper's integrity.

## Declaration of competing interest

The authors affirm that they maintain no associations or engagements with institutions or entities possessing financial or non-financial stakes in the topics or resources examined in this manuscript.
